# Effects of Calcium Nitrate and Magnesium Sulfate Concentrations on Fruit Quality and Physiological Disorders of Musang King Durian (*Durio zibethinus* Murr.) in the Mekong Delta, Vietnam

**DOI:** 10.1155/tswj/1153166

**Published:** 2026-07-30

**Authors:** Nguyen Huynh Duong, Le Vinh Thuc

**Affiliations:** ^1^ Faculty of Crop Science, College of Agriculture, Can Tho University, Can Tho, Vietnam, ctu.edu.vn

**Keywords:** carbohydrate metabolism, fruit physiological disorders, fruit quality, Musang King durian, yield optimization

## Abstract

This study is aimed at determining the optimal foliar application concentrations of calcium nitrate in combination with magnesium sulfate during the fruit development stage in relation to physiological disorders in 7‐year‐old Musang King durian. The experiment was conducted in the Mekong Delta, Vietnam, from November 2024 to April 2025. The experiment was arranged in a two‐factor completely randomized block design. The first factor consisted of four Ca(NO_3_)_2_ concentrations (0%, 0.2%, 0.4%, and 0.6%), applied at 40 DAFS, whereas the second factor included four MgSO_4_ concentrations (0%, 0.2%, 0.4%, and 0.6%), applied at 50 DAFS. Each treatment was replicated four times, with one tree per replication. Spraying Ca(NO_3_)_2_ at a concentration of 0.2%–0.6% increased yield by 4.4%–9.4%. Different concentrations of MgSO_4_ did not significantly affect yield. Foliar application of 0.4% Ca(NO_3_)_2_ combined with 0.2%–0.4% MgSO_4_ did not induce physiological disorders. During the period from fruit development to harvest, nutrient management for Musang King durian can be divided into three soil‐applied NPK fertilization events and two foliar applications of Ca(NO_3_)_2_ and MgSO_4_ to mitigate physiological disorders. NPK fertilizer should be applied at a rate of 0.75 kg tree^−1^ per application, with an N:P:K ratio of 2:1:1 at 30 days after fruit set, 2:1:3 at 45 days after fruit set, and 1:1:3 at 60 days after fruit set, in combination with foliar application of 0.4% Ca(NO_3_)_2_ at 40 days after fruit set and 0.2% MgSO_4_ at 50 days after fruit set.

## 1. Introduction

Durian (*Durio zibethinus* Murr.) is a tropical fruit native to Southeast Asia, belonging to the genus *Durio* within the Malvaceae family, and is widely known as the “king of fruits” due to its distinctive aroma, rich flavor, high nutritional value, and significant economic importance [1]. In recent years, increasing global demand has driven the diversification and improvement of durian cultivars, particularly high‐quality premium varieties [2, 3].

Musang King is regarded as one of the most prestigious durian cultivars worldwide, characterized by its deep yellow pulp, exceptionally high soluble solids content (°Brix exceeding 33% and potentially reaching 39%–44%), creamy texture, and complex aroma profile [4]. Originating from Malaysia, Musang King is a major export cultivar and is often marketed in frozen form due to its susceptibility to fruit cracking and moisture loss at full ripeness [5, 6]. In Vietnam, this cultivar was introduced during 2016–2017, expanded rapidly after 2018, and began commercial fruiting around 2022. However, fruit quality remains inconsistent, largely due to the frequent occurrence of physiological disorders [7].

Physiological disorders in Musang King durian have been reported to include pulp browning, hardened pulp texture, and discoloration. “Hard flesh and discoloration” refers to a condition in which the fruit flesh becomes firm and loses its characteristic yellow color, turning translucent white or opaque white. “Hard flesh without discoloration” causes the edible portion of the fruit to become firm and nonsoft while still retaining its characteristic yellow color. “Aril burn” causes the edible flesh to turn dark brown or black. When aril burn occurs at a low level, it appears only in small spots on the aril; however, when severe, the entire aril may turn black [8]. Similar symptoms were described earlier by Nakasone and Paull [9], who reported uneven coloration and loss of the characteristic bright yellow pulp. These disorders are not caused by pests or pathogens but rather by suboptimal cultivation practices and environmental stresses that alter cellular physiology, ultimately reducing fruit quality and grower profitability [10].

Calcium and magnesium are essential nutrients that play critical roles in fruit development and quality [11, 12]. Calcium is fundamental for maintaining cell wall integrity and membrane stability, regulating ion selectivity, and reducing the activity of cell wall–degrading enzymes [13]. Calcium deficiency often leads to increased membrane permeability and cellular dysfunction due to its limited mobility in the phloem, thereby predisposing fruits to physiological disorders [14]. Magnesium, in contrast, is central to photosynthesis, carbohydrate transport, enzyme activation, and energy metabolism, directly influencing plant growth, yield, and fruit quality [15, 16]. Foliar magnesium application has been shown to enhance protein synthesis, carbohydrate metabolism, enzyme activation, and energy transfer [17]. Moreover, the combined supply of calcium and magnesium has been reported to increase mineral accumulation in fruits, improve yield and quality, and promote starch‐to‐sugar conversion during fruit ripening. Calcium supplementation aims to protect the cell walls of the fruit flesh and maintain cell integrity, while magnesium enhances photosynthetic capacity and promotes starch accumulation during fruit development [18–20].

Therefore, this study is aimed at determining appropriate foliar application rates of calcium and magnesium to mitigate physiological disorders and improving the yield and fruit quality of Musang King durian cultivated in the Mekong Delta.

## 2. Materials and Methods

The experiment was conducted from November 2024 to April 2025 in Tan Binh commune, Can Tho City, Vietnam. A two‐factor factorial experiment was arranged in a randomized complete block design (RCBD) with four replications, in which each experimental unit consisted of a single tree. The first factor consisted of four concentrations of calcium nitrate (Ca(NO_3_)_2_) (0%, 0.2%, 0.4%, and 0.6%), whereas the second factor consisted of four concentrations of magnesium sulfate (MgSO_4_) (0%, 0.2%, 0.4%, and 0.6%). In total, 64 trees were included in the experiment.

All treatments received a uniform basal fertilization rate of 0.75 kg NPK per tree per application. Fertilizer was applied at 30 days after fruit set (DAFS) with an N:P:K ratio of 2:1:1, at 45 DAFS with a ratio of 2:1:3, and at 60 DAFS with a ratio of 1:1:3. The combinations of experimental treatments are presented in Table 1.

**Table 1:** Table of experimental treatment combinations showing the effect of Ca (NO_3_)_2_ and MgSO_4_ concentration on physiological disorders in Musang King durian.

Concentration (%) Ca(NO_3_)_2_–(A)	Concentration (%) MgSO_4_–(B)			
	0 (B_1_)	0.2 (B_2_)	0.4 (B_3_)	0.6 (B_4_)
0 (A_1_)	A_1_B_1_	A_1_B_2_	A_1_B_3_	A_1_B_4_
0.2 (A_2_)	A_2_B_1_	A_2_B_2_	A_2_B_3_	A_2_B_4_
0.4 (A_3_)	A_3_B_1_	A_3_B_2_	A_3_B_3_	A_3_B_4_
0.6 (A_4_)	A_4_B_1_	A_4_B_2_	A_4_B_3_	A_4_B_4_

In Foliar application method, calcium nitrate was applied at 40 DAFS, whereas magnesium sulfate was applied at 50 DAFS for all treatments. Spraying was performed to ensure uniform wetting of both the canopy and fruit surface. Chemicals were sprayed early in the morning under sunny conditions and in the absence of rainfall. Each tree received 10 L of spray solution, prepared according to the concentration assigned to each treatment.

The chemicals and fertilizers used in the experiment are as follows: nitrogen fertilizer (46% N) and phosphate fertilizer (18–46–0) manufactured in Russia; potassium fertilizer in the form of K_2_SO_4_ (50% K_2_O) manufactured in Israel; MgSO_4_ (99%) manufactured in the United Kingdom; Ca(NO_3_)_2_ (99%) manufactured in Norway; and calcium (5%) and boron in the form of H_3_BO_3_ manufactured in Germany. The NPK formulation with a ratio of 2:1:1 contained 20% N, 10% P_2_O_5_, and 10% K_2_O; the 2:1:3 formulation contained 20% N, 10% P_2_O_5_, and 30% K_2_O; and the 1:1:3 formulation contained 10% N, 10% P_2_O_5_, and 30% K_2_O.

Leaf and soil samples were collected at harvest. Leaf samples were taken from the second to third fully expanded leaves from the outer canopy toward the interior for each treatment (50 leaves were collected from each tree, evenly distributed around the tree canopy), oven‐dried at 60°C, and finely ground prior to analysis. Soil samples were collected at a depth of 20 cm using a specialized soil auger; four subsamples were taken per tree and composited into a single sample (analysis was conducted on each individual tree). Soil and leaf samples were analyzed for total nitrogen, total phosphorus, available phosphorus, exchangeable potassium, and organic matter [21]. Total potassium was determined by atomic absorption spectrophotometry at a wavelength of 766.5 nm, calcium at 422.5 nm, and magnesium at 285.2 nm.

Five fruits per tree were harvested for analysis when the first fruit on the tree reached maturity. Fruit samples were transported to the laboratory and allowed to ripen naturally at room temperature (28°C–30°C). After 2–3 days, when ripening commenced, quality analyses were conducted. Fruit weight was determined by weighing five fruits per tree and calculating the mean value. The proportions of peel weight, pulp weight, and seed weight were calculated as percentages of total fruit weight. Fruit yield per tree (kg tree^−1^) was calculated as the average fruit weight multiplied by the number of fruits per tree, whereas theoretical yield (t ha^−1^) was estimated by multiplying yield per tree by 200 trees ha^−1^ and dividing by 1000 to convert kilograms to tons. Starch content was determined according to Comb et al. [22], total sugar content by the phenol–sulfuric acid method [23], soluble solids content (°Brix) using an Atago refractometer, moisture content as (1 − dry sample weight/fresh weight) × 100, the proportion of pulp affected by physiological disorders as (number of affected segments/total number of segments) × 100, the proportion of hardened and discolored pulp, the proportion of hardened pulp, and the proportion of pulp burn.

The data were subjected to analysis of variance (ANOVA) to detect significant differences among treatment means, and mean comparisons were performed using Duncan′s multiple range test.

## 3. Results and Discussion

### 3.1. Leaf Nutrient Contents

The concentrations of nutrients in leaves differed significantly at the 1% significance level among the different Ca(NO_3_)_2_ concentrations (Table 2). Foliar application of Ca(NO_3_)_2_ at a concentration of 0.6% resulted in significantly lower total nitrogen (2.03%), total phosphorus (0.15%), and total potassium (1.63%) contents compared with the other concentrations. In contrast, calcium and magnesium contents increased with increasing Ca(NO_3_)_2_ concentration, indicating that higher Ca(NO_3_)_2_ application rates enhanced the accumulation of these elements in leaves. Foliar application of MgSO_4_ at different concentrations did not significantly affect total phosphorus, total potassium, or calcium contents; however, total nitrogen content differed significantly at the 5% level, and magnesium content differed significantly at the 1% level.

**Table 2:** Effects of Ca(NO_3_)_2_ and MgSO_4_ concentrations on leaf nutrient contents after the experiment.

Treatment	Total N (%)	Total *p*(%)	Total K (%)	Ca (%)	Mg (%)
Concentration (A) Ca(NO_3_)_2_ (%)					
0	2.25^a^	0.20^a^	1.81^a^	1.69^c^	0.28^c^
0.2	2.18^b^	0.19^ab^	1.77^b^	1.87^b^	0.34^b^
0.4	2.11^c^	0.18^b^	1.69^c^	1.90^b^	0.37^a^
0.6	2.03^d^	0.15^c^	1.63^d^	2.00^a^	0.38^a^
Concentration (B) MgSO_4_ (%)					
0	2.16^a^	0.18	1.74	1.87	0.28^d^
0.2	2.16^a^	0.18	1.73	1.86	0.32^c^
0.4	2.13^b^	0.18	1.72	1.87	0.36^b^
0.6	2.15^ab^	0.18	1.71	1.87	0.40^a^
Significant differences					
(A)	^∗∗^	^∗∗^	^∗∗^	^∗∗^	^∗∗^
(B)	^∗^	*ns*	*ns*	*ns*	^∗∗^
(A ^∗^B)	*ns*	*ns*	*ns*	*ns*	^∗∗^
C.V. (%)	1.40	7.34	1.52	2.38	5.44

*Note:* Within a column, means followed by the same letter are not significantly different according to Duncan′s multiple range test.

Abbreviation: ns, not significant.

^∗^significant at *p* ≤ 0.05.

^∗∗^significant at *p* ≤ 0.01.

Wilkinson et al. [24] reported that nitrogen enhances phosphorus uptake in plants by promoting root growth, increasing phosphorus transport in roots, and lowering soil pH through the uptake of NH_4_^+^, thereby increasing phosphorus solubility. Potassium nutrition exhibits antagonistic interactions with calcium and magnesium when present at high concentrations, which is attributed to the high mobility of potassium. Excessive potassium application leads to competition among cations such as K^+^, Ca^2+^, and Mg^2+^, which may induce magnesium and calcium deficiencies and disrupt ion exchange within plants. According to Li et al. [25], magnesium concentration does not affect potassium content during the early growth stage; however, a significant negative correlation in leaves and a positive correlation in fruits was observed during the later growth stage. During translocation from roots to aboveground organs, magnesium and calcium are mainly distributed in leaves, whereas potassium is preferentially allocated to fruits. Therefore, supplying calcium and magnesium via direct foliar application to fruits is necessary to meet fruit nutrient demands and improve fruit quality.

Optimal leaf nutrient concentration ranges are reported as 2.0%–2.4% for total nitrogen, 0.15%–0.25% for total phosphorus, 1.5%–2.5% for total potassium, 1.7%–2.5% for calcium, and 0.25%–0.50% for magnesium [26]. Studies on nutrient correlations in durian leaves by Lim and Luders [27] indicated a positive correlation between nitrogen and potassium contents, whereas nitrogen and calcium showed a negative correlation [28]. According to Tariq et al. [29], phosphorus enhances the uptake of nitrogen and potassium. The antagonistic relationship between potassium and calcium in leaves is stronger than that between potassium and magnesium, while calcium exhibits a positive correlation with magnesium [30]. Tian et al. [31] reported that magnesium is a key element influencing biochemical and physiological processes in plants. In addition, magnesium stimulates and enhances the uptake of nutrients such as nitrogen, phosphorus, potassium, and calcium during both vegetative growth and reproductive stages [32]. Therefore, adequate nutrient supplementation plays an important role in mitigating physiological disorders in durian.

### 3.2. Calcium and Magnesium Content in the Fruit Pulp

Calcium and magnesium contents in the fruit pulp differed significantly at the 1% significance level among the different concentrations of Ca(NO_3_)_2_ and MgSO_4_, with no interaction observed between the two factors (Table 3). Foliar application of Ca(NO_3_)_2_ at a concentration of 0.6% resulted in the highest calcium content in the fruit pulp (10.7 mg 100 g^−1^), which was significantly higher than those obtained at the other concentrations. Magnesium content at this concentration (31.2 mg 100 g^−1^) did not differ significantly from that obtained with Ca(NO_3_)_2_ at 0.4%, but was significantly higher than that of the control and the 0.2% treatment. Similarly, foliar application of MgSO_4_ at 0.6% resulted in the highest magnesium content in the fruit pulp (31.2 mg 100 g^−1^), which differed significantly from the other concentrations.

**Table 3:** Effects of Ca(NO_3_)_2_ and MgSO_4_ concentrations on nutrient contents in the pulp of Musang King durian after the experiment.

Treatment	Ca (mg/100 g)	Mg (mg/100 g)
Concentration (A) Ca(NO_3_)_2_ (%)		
0	8.8^c^	27.8^c^
0.2	9.5^b^	29.1^b^
0.4	9.8^b^	30.7^a^
0.6	10.7^a^	31.2^a^
Concentration (B) MgSO_4_ (%)		
0	9.3^c^	27.5^d^
0.2	9.6^bc^	29.1^c^
0.4	9.8^ab^	30.5^b^
0.6	10.0^a^	31.7^a^
Significant differences		
(A)	^∗∗^	^∗∗^
(B)	^∗∗^	^∗∗^
(A ^∗^B)	*ns*	*ns*
C.V. (%)	4.10	3.13

*Note:* Within a column, means followed by the same letter are not significantly different according to Duncan′s multiple range test.

Abbreviation: ns, not significant.

^∗∗^significant at *p* ≤ 0.01.

Previous studies on fruit ripening of the Monthong cultivar have shown that calcium content decreased from 11.3 mg 100 g^−1^ at physiological maturity to 8.4 mg 100 g^−1^ at full ripeness, whereas magnesium content declined slightly from 34.1 to 29.6 mg 100 g^−1^, indicating mineral dilution associated with increased dry matter accumulation in the fruit pulp [33]. Research conducted in Malaysia has also reported substantial variation in calcium and magnesium contents among durian cultivars, with calcium ranging from 6.5 to 13.2 mg 100 g^−1^ and magnesium from 22.8 to 34.6 mg 100 g^−1^, reflecting the strong influence of genetic factors and soil conditions on mineral accumulation in the fruit pulp [34].

### 3.3. Fruit Weight and Fruit Weight Components

Fruit weight differed significantly among the different Ca(NO_3_)_2_ concentrations, whereas no significant differences were observed among the MgSO_4_ concentrations, and no interaction between the two factors was detected (Table 4). Foliar application of Ca(NO_3_)_2_ at a concentration of 0.6% produced the highest fruit weight (2236 g), which did not differ significantly from that obtained at 0.4% Ca(NO_3_)_2_ but was significantly higher than those recorded at 0.2% Ca(NO_3_)_2_ and the control treatment. In contrast, the proportions of fruit weight components were not significantly affected by either Ca(NO_3_)_2_ or MgSO_4_ concentrations, and no interaction between the two factors was observed. During the main cropping season, the proportions of peel weight, pulp weight, and seed weight averaged 70.4%, 24.7%, and 4.9%, respectively. Calcium plays a critical role in maintaining cell turgor pressure throughout the fruit ripening process [35], thereby contributing to increased peel mass and, consequently, greater overall fruit weight [36].

**Table 4:** Effects of Ca(NO_3_)_2_ and MgSO_4_ concentrations on fruit weight components of Musang King durian after the experiment.

Treatment	Fruit weight (g)	Peel weight (%)	Pulp weight (%)	Seed weight (%)
Concentration (A) Ca(NO_3_)_2_ (%)				
0	2032^c^	70.2	24.9	4.9
0.2	2118^b^	70.4	24.6	5.0
0.4	2152^ab^	70.5	24.5	5.0
0.6	2236^a^	70.6	24.6	4.8
Concentration (B) MgSO_4_ (%)				
0	2118	70.4	24.8	4.8
0.2	2130	70.2	24.9	4.9
0.4	2140	70.5	24.6	4.9
0.6	2150	70.6	24.5	4.9
Significant differences				
(A)	^∗∗^	*ns*	*ns*	*ns*
(B)	*ns*	*ns*	*ns*	*ns*
(A ^∗^B)	*ns*	*ns*	*ns*	*ns*
C.V. (%)	2.40	1.10	0.53	5.45

*Note:* Within a column, means followed by the same letter are not significantly different according to Duncan′s multiple range test.

Abbreviation: ns, not significant.

^∗∗^significant at *p* ≤ 0.01.

### 3.4. Yield

The number of fruits per tree did not differ significantly among the different Ca(NO_3_)_2_ or MgSO_4_ concentrations, with an average of 41.3 fruits tree^−1^. The lack of significant differences among treatments was attributed to fruit thinning applied uniformly across all experimental treatments. Fruit yield and theoretical yield differed significantly at the 1% significance level among the Ca(NO_3_)_2_ concentrations, whereas no significant differences were observed among the MgSO_4_ concentrations, and no interaction between the two factors was detected (Table 5). Foliar application of Ca(NO_3_)_2_ at a concentration of 0.6% resulted in a yield of 91.8 kg tree^−1^, which did not differ significantly from yields obtained at 0.2% and 0.4% Ca(NO_3_)_2_ (87.6 and 88.8 kg tree^−1^, respectively), but was significantly higher than that of the control treatment without Ca(NO_3_)_2_ application. Foliar application of MgSO_4_ at different concentrations did not significantly affect yield, with an average yield of 88.1 kg tree^−1^ across treatments. Yield is the product of fruit weight and number of fruits per tree. Although the number of fruits per tree did not differ significantly according to statistical analysis, fruit weight showed a significant difference, leading to a significant difference in overall yield. This indicates that foliar application of Ca(NO_3_)_2_ has an effect on the yield of Musang King durian, whereas MgSO_4_ application does not increase yield.

**Table 5:** Effects of Ca(NO_3_)_2_ and MgSO_4_ concentrations on yield of Musang King durian.

Treatment	Fruits tree^−1^	Yield (kg tree^−1^)	Theoretical yield (t ha^−1^)
Concentration (A) Ca(NO_3_)_2_ (%)			
0	41.3	83.9^b^	16.8^b^
0.2	41.4	87.6^ab^	17.5^ab^
0.4	41.3	88.8^ab^	17.8^ab^
0.6	41.1	91.8^a^	18.4^a^
Concentration (B) MgSO_4_ (%)			
0	41.1	87.0	17.4
0.2	41.3	88.0	17.6
0.4	41.3	88.3	17.7
0.6	41.4	88.9	17.8
Significant differences			
(A)	*ns*	^∗∗^	^∗∗^
(B)	*ns*	*ns*	*ns*
(A∗B)	*ns*	*ns*	*ns*
C.V. (%)	5.48	5.65	5.65

*Note:* Within a column, means followed by the same letter are not significantly different according to Duncan′s multiple range test.

Abbreviation: ns, not significant

^∗∗^significant at *p* ≤ 0.01.

### 3.5. Fruit Quality of Musang King Durian

Total sugar content differed significantly among the different Ca(NO_3_)_2_ concentrations at the 1% significance level and among the MgSO_4_ concentrations at the 5% significance level, with a significant interaction between the two factors at the 1% level (Table 6). Treatments without Ca(NO_3_)_2_ application exhibited a total sugar content of 11.5%, which was significantly lower than that of treatments receiving Ca(NO_3_)_2_. Similarly, foliar application of MgSO_4_ at concentrations ranging from 0.2% to 0.6% resulted in significantly higher total sugar content compared with treatments without MgSO_4_ application. Starch content also differed significantly at the 1% significance level among the different Ca(NO_3_)_2_ and MgSO_4_ concentrations, and a significant interaction between the two factors was observed (Table 7). The combined application of 0.4% Ca(NO_3_)_2_ and 0.2% MgSO_4_ produced a high total sugar content (13.6%), which did not differ significantly from other combined treatments involving Ca(NO_3_)_2_ (0.2%–0.6%) and MgSO_4_ (0.2%–0.6%), but was significantly higher than treatments in which only one compound was applied without the other at the 1% significance level. Treatments without Ca(NO_3_)_2_ and MgSO_4_ application exhibited high starch content and low sugar content during fruit ripening. In contrast, foliar application of Ca(NO_3_)_2_ at 0.4%–0.6% combined with MgSO_4_ at 0.2%, or Ca(NO_3_)_2_ at 0.6% combined with MgSO_4_ at 0.4%, resulted in significantly lower starch content compared with treatments receiving Ca(NO_3_)_2_ alone or MgSO_4_ alone.

**Table 6:** Effects of Ca(NO_3_)_2_ and MgSO_4_ concentrations on total sugar and starch contents in the pulp of Musang King durian after the experiment.

Treatment	Concentration (B) MgSO_4_ (%)	Concentration (A) Ca(NO_3_)_2_ (%)				Mean B
		0	0.2	0.4	0.6	
Total sugar (%)	0	11.4^b^	12.0^b^	11.9^b^	11.4^b^	11.7^B^
	0.2	11.7^b^	12.9^ab^	13.6^a^	13.0^ab^	12.8^A^
	0.4	11.6^b^	12.9^ab^	12.7^ab^	12.9^ab^	12.5^A^
	0.6	11.4^b^	13.0^ab^	13.1^ab^	12.9^ab^	12.6^A^
	Mean (A)	11.5^B^	12.7^A^	12.8^A^	12.5^A^	
	Significance A	^∗∗^				
	Significance B	^∗^				
	Significance A ^∗^B	^∗∗^				
	C.V. (%)	6.59				
Starch (%)	0	13.9^a^	13.6^a^	13.7^a^	13.7^a^	13.7^B^
	0.2	13.7^a^	13.1^ab^	11.7^b^	12.5^b^	12.7^A^
	0.4	13.7^a^	12.6^ab^	12.0^ab^	11.3^b^	12.4^A^
	0.6	13.9^a^	12.4^ab^	12.0^ab^	13.1^a^	12.9^A^
	Mean (A)	13.8^A^	12.9^B^	12.4^B^	12.7^B^	
	Significance A	^∗∗^				
	Significance B	^∗∗^				
	Significance A ^∗^B	^∗∗^				
	C.V. (%)	6.68				

*Note:* Within a column, means followed by the same letter are not significantly different according to Duncan′s multiple range test.

^∗^significant at *p* ≤ 0.05.

^∗∗^significant at *p* ≤ 0.01.

**Table 7:** Effects of Ca(NO_3_)_2_ and MgSO_4_ concentrations on the proportion of physiological disorders relative to total observed samples and the proportion of affected pulp segments in Musang King durian.

Treatment	Concentration (B) MgSO_4_ (%)	Concentration (A) ca(NO_3_)_2_ (%)				Mean B
		0	0.2	0.4	0.6	
Affected fruits (%)	0	30.0^a^	20.0^ab^	15.0^abc^	15.0^abc^	20.0^A^
	0.2	30.0^a^	5.0^bc^	0.0^d^	5.0^bc^	10.0^B^
	0.4	25.0^ab^	5.0^bc^	0.0^d^	5.0^bc^	8.8^B^
	0.6	20.0^ab^	5.0^bc^	10.0^bc^	10.0^bc^	11.3^B^
	Mean (A)	26.3^A^	8.8^B^	6.3^B^	8.8^B^	
	Significance A	^∗∗^				
	Significance B	^∗∗^				
	Significance A ^∗^B	^∗∗^				
	CV (%)	63.3				
Disordered segments (%)	0	29.9^a^	28.4^a^	14.2^ab^	21.1^ab^	23.4^A^
	0.2	27.5^a^	2.1^cd^	0.0^d^	4.2^bcd^	8.4^B^
	0.4	20.5^ab^	6.3^bcd^	0.0^d^	3.9^bcd^	7.7^B^
	0.6	30.5^a^	4.6^bcd^	5.9^bcd^	4.4^bcd^	11.3^B^
	Mean (A)	27.1^A^	10.3^B^	5.0^B^	8.4^B^	
	Significance A	^∗∗^				
	Significance B	^∗∗^				
	Significance A ^∗^B	^∗^				
	CV (%)	60.4				

*Note:* Within a column, means followed by the same letter are not significantly different according to Duncan′s multiple range test.

^∗^significant at *p* ≤ 0.05.

^∗∗^significant at *p* ≤ 0.01.

During fruit development, starch accumulation in durian fruit increases and subsequently decreases markedly as starch is converted into sugars during ripening [37]. As starch degradation progresses and soluble sugar content increases, the fruit pulp transitions from a firm texture to a softer consistency, accompanied by increased sweetness. This process is a key determinant of sensory quality [38]. The increase in soluble sugars during ripening enhances sweetness and pulp texture, thereby improving overall durian fruit quality [39]. Sugar translocation from leaves to fruits is proportional to starch degradation mediated by enzymes [40] such as ADP‐glucose pyrophosphorylase (AGPase) and amylases during fruit ripening [41]. The activity of extracellular invertases, together with interactions involving water status and nutrient balance, particularly nitrogen, potassium, and calcium, forms a complex regulatory network in which disruption of any component may lead to physiological disorders [42].

**Figure 1:**
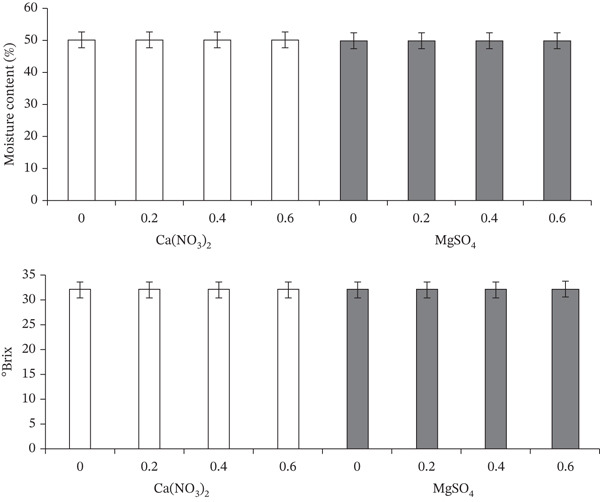
Effects of Ca(NO_3_)_2_ and MgSO_4_ concentrations on moisture content and soluble solids (°Brix) of Musang King durian fruit.

Moisture content (49.8%) and soluble solids content (32.2°Brix) of Musang King durian did not differ significantly at the 5% significance level among the different Ca(NO_3_)_2_ and MgSO_4_ concentrations, and no interaction between the two factors was observed (Figure 1). According to Belgia [43], the moisture content of durian pulp typically ranges from 49% to 59%, which is consistent with the values observed in the present study.

### 3.6. Physiological Disorders

The proportion of physiological disorders relative to the total number of observed samples and the proportion of pulp segments affected by physiological disorders differed significantly at the 1% significance level among the different Ca(NO_3_)_2_ and MgSO_4_ concentrations, and a significant interaction between the two factors was also observed (Table 7). Foliar application of Ca(NO_3_)_2_ at concentrations ranging from 0.2% to 0.6%, or application of MgSO_4_ alone at concentrations of 0.2%–0.6%, resulted in significantly lower proportions of physiological disorders and affected pulp segments compared with the control treatment without foliar application. The combined application of 0.4% Ca(NO_3_)_2_ at 40 DAFS and 0.2%–0.4% MgSO_4_ at 50 DAFS resulted in no occurrence of physiological disorders and differed significantly from other Ca(NO_3_)_2_ and MgSO_4_ combinations at the 1% significance level for the proportion of physiological disorders relative to total observed samples and at the 5% significance level for the proportion of affected pulp segments.

Magnesium deficiency reduces total carbohydrate production and limits the translocation of sugars from leaves to fruits, leading to reduced pulp quality, increased susceptibility to physiological disorders, or restricted fruit growth. In addition, magnesium plays an important role in maintaining nutrient balance with potassium and calcium, and appropriate K:Mg or Ca:Mg ratios can alleviate physiological stress in plants [44].

**Figure 2:**
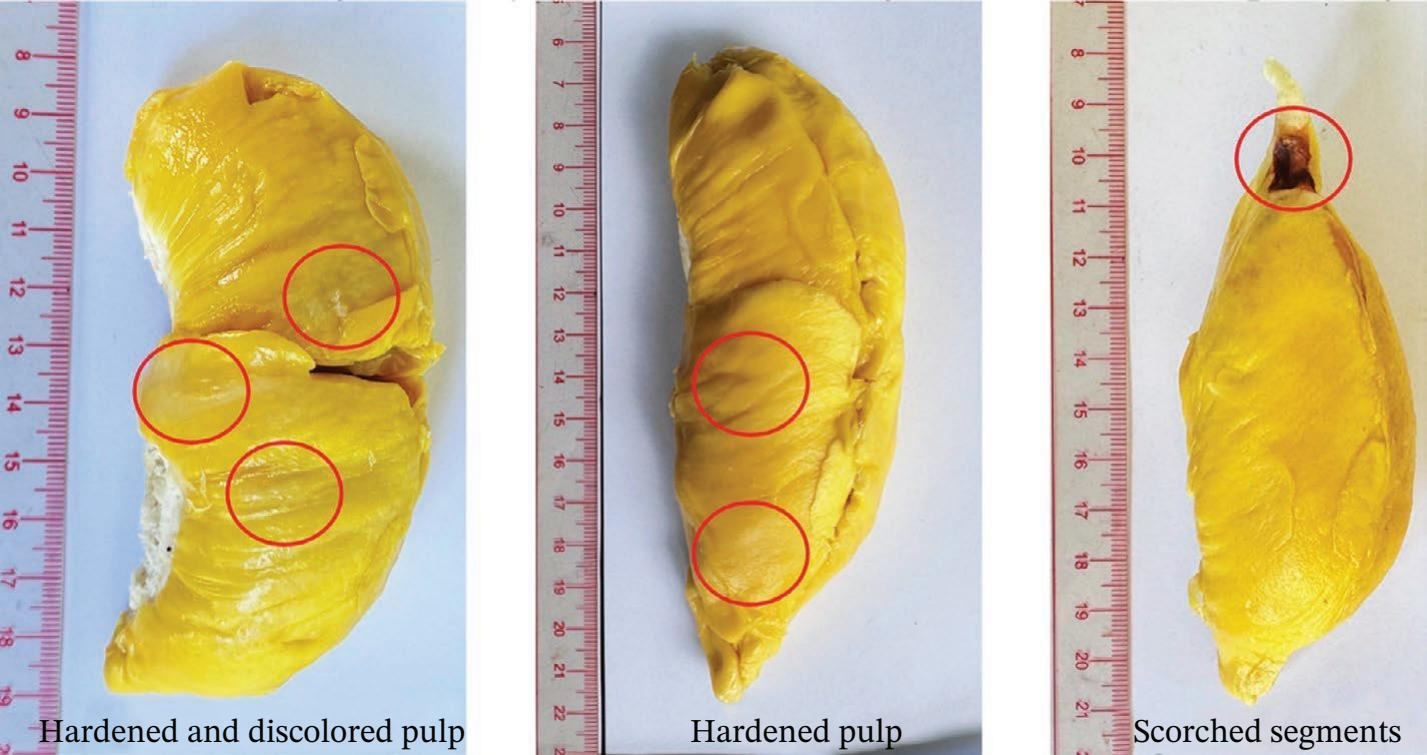
Physiological disorders in Musang King durian.

The statistical analysis presented in Table 8 and Figure 2 indicates that the proportion of hardened pulp with discoloration differed significantly at the 1% significance level among the different Ca(NO_3_)_2_ and MgSO_4_ concentrations, with no interaction between the two factors. Treatments without Ca(NO_3_)_2_ application or without MgSO_4_ application exhibited a high proportion of hardened and discolored pulp (13.3%), which was significantly higher than that observed in all other treatments. The proportion of hardened pulp also differed significantly at the 1% significance level among the different Ca(NO_3_)_2_ and MgSO_4_ concentrations. Foliar application of Ca(NO_3_)_2_ (0.2%–0.6%) or MgSO_4_ (0.2%–0.6%) resulted in a significantly lower proportion of hardened pulp without discoloration compared with treatments without Ca(NO_3_)_2_ or MgSO_4_ application. Foliar application of Ca(NO_3_)_2_ at 0.4% resulted in no pulp burn, with no significant difference compared with applications at 0.2% and 0.6%.

**Table 8:** Effects of Ca(NO_3_)_2_ and MgSO_4_ concentrations on physiological disorders of Musang King durian.

Treatment	Hardened and discolored pulp (%)	Hardened pulp (%)	Scorched segments (%)
Concentration (A) Ca(NO_3_)_2_ (%)			
0	13.3^a^	10.2^a^	3.6^a^
0.2	5.7^b^	3.1^b^	1.0^ab^
0.4	3.1^b^	2.0^b^	0.0^b^
0.6	4.4^b^	3.0^b^	1.0^b^
Concentration (B) MgSO_4_ (%)			
0	12.7^a^	7.1^a^	3.5^a^
0.2	4.5^b^	3.5^b^	0.5^b^
0.4	4.1^b^	3.0^b^	0.5^b^
0.6	5.7^b^	4.7^b^	1.0^b^
Significant differences			
(A)	^∗∗^	^∗∗^	^∗∗^
(B)	^∗∗^	^∗∗^	^∗∗^
(A ^∗^B)	*ns*	*ns*	*ns*
C.V. (%)	48.8	48.5	64.5

*Note:* Within a column, means followed by the same letter are not significantly different according to Duncan′s multiple range test.

Abbreviation: ns, not significant

^∗∗^significant at *p* ≤ 0.01.

Magnesium is distributed within the cytoplasm, chloroplasts, and vacuoles and directly participates in the activity of enzymes involved in carbohydrate synthesis and energy metabolism [45]. The distribution and bioavailability of calcium in fruit pulp tissues directly determine cell wall integrity, water‐holding capacity, and the mechanical strength of pulp segments, thereby influencing typical physiological disorders such as hardened pulp, softened pulp, pulp burn, and fruit cracking [46, 47]. Magnesium in fruit pulp cells affects starch biosynthesis, sugar–starch balance, reserve accumulation, and sugar distribution among pulp segments [48]. Magnesium deficiency reduces ATP‐dependent enzyme efficiency and metabolic phosphorylation flexibility, thereby decreasing starch synthesis rates and increasing the risk of pulp burn and hardened pulp disorders [49, 50]. The significant increase in Ca and Mg contents in the fruit flesh (Table 3) reduced physiological disorder symptoms.

Ultimately, reducing physiological disorders through magnesium management requires an integrated strategy, including periodic assessment of soil and leaf magnesium status, optimization of N:P:K:Mg ratios, and irrigation management to maintain xylem flow [48]. When rapid correction is required, foliar magnesium application should be implemented, together with calcium and micronutrient supplementation (boron and zinc) to support cell wall structure and enzymatic activity. Field‐scale trials have demonstrated that such integrated management strategies reduce fruit cracking and uneven ripening, increase soluble solids content in the pulp, and improve overall commercial quality [51, 52]. Therefore, effective calcium and magnesium supply plays a crucial role in mitigating physiological disorders in Musang King durian fruit.

The significant increase in Ca and Mg contents in the fruit flesh reduced physiological disorder symptoms.

Accordingly, in Musang King durian cultivation in the Mekong Delta, the application of NPK fertilizer at a ratio of 2:1:1 at 30 days, 2:1:3 at 45 days, and 1:1:3 at 60 DAFS, at a rate of 0.75 kg tree^−1^ per application, during both the main and off seasons, can optimize yield while effectively reducing the incidence of physiological disorders. In addition, foliar application of 0.4% Ca(NO_3_)_2_ combined with 0.2% MgSO_4_ results in optimal yield performance and prevents the occurrence of physiological disorders in Musang King durian fruit.

## 4. Conclusion

During the period from fruit development to harvest, nutrient management for Musang King durian can be divided into three soil‐applied NPK fertilization events and two foliar applications of Ca(NO_3_)_2_ and MgSO_4_ to mitigate physiological disorders. NPK fertilizer should be applied at a rate of 0.75 kg tree^−1^ per application, with an N:P:K ratio of 2:1:1 at 30 DAFS, 2:1:3 at 45 DAFS, and 1:1:3 at 60 DAFS, in combination with foliar application of 0.4% Ca(NO_3_)_2_ at 40 DAFS and 0.2% MgSO_4_ at 50 DAFS. Further studies involving similar experiments under different locations and cropping seasons are required.

## Author Contributions

The two authors contributed equally.

## Funding

This work was supported by the Department of Science and Technology of Hau Giang Province, No. 39/HD‐KHCN.

## Disclosure

The authors accepted the responsibility for the content of the manuscript and consented to its submission, reviewed all the results, and approved the final version of the manuscript.

## Conflicts of Interest

The authors declare no conflicts of interest.

## Data Availability

The data that support the findings of this study are available from the corresponding author upon reasonable request.
